# Measuring awareness in people with dementia: protocol for a scoping review

**DOI:** 10.1186/s13643-019-1078-5

**Published:** 2019-07-04

**Authors:** Catherine M. Alexander, Anthony Martyr, Sharon A. Savage, Linda Clare

**Affiliations:** 1 REACH: The Centre for Research in Ageing and Cognitive Health, College of Medicine and Health, University of Exeter, South Cloisters, St Luke’s Campus, Exeter, EX1 2LU UK; 2 REACH: The Centre for Research in Ageing and Cognitive Health, School of Psychology, University of Exeter Medical School and College of Life and Environmental Sciences, St Luke’s Campus, Exeter, EX1 2LU UK; 3 Psychology, College of Life and Environmental Sciences, University of Exeter, Washington Singer Laboratories, Streatham Campus, Exeter, EX4 4QG UK

**Keywords:** Alzheimer’s, Unawareness, Anosognosia, Insight, Metacognition, Discrepancy, Denial

## Abstract

**Background:**

People with dementia (PwD) vary in the degree of awareness they show about their situation, both generally concerning the diagnosis and more specifically around certain aspects or objects of awareness such as awareness of memory impairment, altered daily activities or social functioning. The extent of awareness or lack of awareness has consequences for well-being of PwD and carers, impacting on rates of hospital admission, institutionalization, mood, adjustment to diagnosis, outcomes from intervention and carer burden. An accurate estimation of a person’s awareness could therefore be useful in a clinical setting to support PwD and their carers in making appropriate choices for health and care decisions, and could facilitate safe management by health care professionals, e.g. in an acute care setting. There is a range of different approaches to measuring awareness reported in the dementia research literature, with varying estimates of the frequency of lack of awareness, reflecting different methodologies and populations. The majority of the methods have been developed for research purposes and may not be suitable for clinical use. There are no recent scoping or systematic reviews of the available methods.

**Method:**

We will conduct a scoping review of published studies that have assessed awareness in people with dementia of all types, and all degrees of severity. The systematic search will include the electronic databases PubMed, Embase, PsycInfo, CINAHL, Web of Science and Cochrane Library, using search terms for dementia (“dement*” or “Alzheimer*” or “Pick’s disease”) and “awareness”, “unawareness”, “anosognosia”, “insight”, “denial”, “metacognit*” or “discrepanc*” identified from pilot searches. Findings will be mapped and described according to the method used, the setting and diagnosis and the object of awareness studied if specified. Validated measures will be identified.

**Discussion:**

This scoping review will provide an overview of the methods used to measure awareness in people with dementia, allowing comparison of the methods along with identification of validated measures. The methods or components will be appraised for potential clinical use, and gaps in research will be highlighted.

**Electronic supplementary material:**

The online version of this article (10.1186/s13643-019-1078-5) contains supplementary material, which is available to authorized users.

## Background

A diagnosis of dementia (or major neurocognitive disorder) is made from the history and observation of acquired cognitive impairment, with a decline in functioning [1], or a decline in mental ability severe enough to interfere with independence and daily life [2]. People with dementia (PwD) show varying degrees of recognition or acknowledgement of impairment or of the resulting changes in their abilities. Estimates of the number of PwD who lack awareness of their difficulties range from 20 to 81%, depending on how it is measured, the degree of dementia severity and in which cultural or geographical population it is measured [3, 4].

Awareness is an important concept for discussions around optimising support for PwD, for several reasons. Lack of awareness of personal limitations is a predictor of risk of unsafe behaviour, hospital admission and institutionalization for PwD [5]. Conversely, some PwD underestimate their abilities, for example, in the more complex realm of social and emotional functioning [6], which might lead to unnecessary avoidance of activities that could be safely managed and enjoyed. In some situations, PwD may be more accurate than carers at appraising their abilities in functional tasks [7], which again could result in conflict or inappropriate restrictions. Lack of awareness of performance or functioning is associated with increased stress for the carer [8, 9] and poorer perceived quality of relationships with the person with dementia [10] which may indirectly impact negatively on quality of life for PwD [11].

Accurate measurement of awareness could lead to a better understanding of the individual’s experience, and a tailored approach to planning of everyday care and activities. Awareness is not routinely assessed in the diagnosis or structured reviews of dementia, but this could be beneficial in a healthcare setting, possibly enabling improved communication and involvement in personal health care decisions. Appreciating the unique perspective of the person with dementia would facilitate health care professionals and family carers in offering person-centred care. Higher awareness has been associated with better outcomes of cognitive rehabilitation [12], and ratings could be used to select appropriate interventions. Ability to measure changes in awareness over time would also be relevant for care reviews or perhaps in assessing the impact of interventions. Distinct from a judgement of capacity which is based on sufficient memory and understanding to make a specific decision at a point in time, information about a person’s awareness might help in an acute care setting. For example, understanding a person’s level of awareness may assist in management decisions around home discharge or self-administration of medication or could contribute in later-stage dementia to “best interest” decisions.

The literature includes a range of terms to describe awareness and similar, related concepts derived from differing theoretical models. “Anosognosia” was initially used to define a specific neurological deficit [13], but has now been broadened to describe a lack of awareness of deficits in different conditions, including dementia, and over a range of domains [14]. “Insight”, originating from psychiatry, is used sometimes interchangeably with awareness, or sometimes as the wider awareness of having a clinical condition and its implications [15]. “Denial” of symptoms or diagnosis suggests psychological factors and personality traits influencing acceptance of the condition [16] and can present as a lack of awareness in dementia. “Metacognition” has been used to describe self-awareness [17] and implies self-reflection, which may lead to acknowledgement of the condition and its impact. Awareness has sometimes been measured as a global construct but ideally should be construed as meaning awareness of something, which can be described as an “object” of awareness, e.g. awareness of memory problems, of functioning in daily activities or of physical symptoms, or of social and emotional functioning [15]. The object should be specified in research studies as it influences the degree of awareness elicited [6]. Awareness of different objects may operate at different “levels”, i.e. registering and responding to basic sensory information, monitoring performance as it takes place, making evaluative judgements or making general appraisals of the situation [18]. Awareness can also be implied through observed responses and behaviour rather than explicitly shown, although few studies have measured this [19, 20].

We will use the term “awareness” as a broad and neutral term encompassing the phenomena, or clinical manifestations, uniting these concepts. We define this as “the ability to hold a reasonable or realistic perception or appraisal of, and/or respond accordingly to, a given aspect of one’s environment, situation, functioning or performance” [21] p., 20. This description allows for discussion of retained awareness or reduced awareness, as well as accommodation of cognitive models which include explicit and implicit awareness [20]. It also supports the idea of neurological and psychosocial influences, with awareness across a range of different objects [22] and at different levels [18].

A range of methods have been devised to measure awareness in dementia, mainly in research settings, with a minority developed specifically for clinical use, for example, the Structured Interview for Insight and Judgment in Dementia [5] and the Abridged Anosognosia Questionnaire [23]. Both quantitative and qualitative research approaches have been used, mainly with people with early-stage dementia. Some observational techniques are particularly suited to measuring awareness in people with more severe dementia, in a residential setting [24]. Quantitative measures of awareness are typically based on either ratings from clinician interviews [25] or questionnaires measuring a discrepancy between participant and informant ratings [26], or the discrepancy between self-rating and performance on a cognitive or functional task [7]. Different judgements may be required by the participants, e.g. reporting severity of problems, frequency of problems and changes in abilities such as no longer managing finances or cooking, over different durations of time or in comparison to healthy individuals. The resultant score may be presented on a scale of awareness [27] or as a dichotomous result, i.e. aware or unaware [28]. Multi-dimensional measures of awareness have been used to provide comprehensive information [8] but are lengthy and would be unsuitable for use in a clinical setting. Other methods have been developed to measure awareness in different populations, e.g. post-traumatic head injury, but may not be appropriate for use in a typically older age group with a progressive condition such as dementia.

To fully appraise the most appropriate methods for measuring awareness in a clinical setting, it would be helpful to have an overview of the approaches taken across the range of settings, and with people at differing stages of dementia. A preliminary search of the literature finds older reviews of methods and measures [29], and more recent systematic reviews that look at different aspects of awareness, e.g. concepts of awareness in dementia [30], more narrowly defined reviews [4, 31] and narrative reviews [17, 32]. To date, we have found no recent comprehensive systematic reviews or scoping reviews in English, of methods of measuring awareness in dementia.

We will therefore conduct a scoping review to map the literature concerning original studies that measure awareness in people with dementia. This will give an overview of the breadth of methods used and allow exploration of the characteristics and utility of the methods. A scoping method has been chosen as, whilst conducted in a rigorous and systematic way, it allows flexibility to include quantitative and qualitative studies, as well as case studies, which reflect clinical and research practice for assessing awareness. We will also identify the validated measures in use, allowing further appraisal of these methods and components, in consideration of potential clinical use. We will follow the Preferred Reporting Items for Systematic Reviews and Meta-Analyses (PRISMA) guidelines for conducting the review and preparing the report [33–35].

## Methods/design

### Objectives

This scoping review seeks to answer the question “What methods and measures are used to assess awareness in people with dementia, and what are the characteristics and utility of each method?” We will systematically scope the literature, following the framework outlined initially by Arksey and O’Malley [36], with further guidance from later publications [35, 37, 38]. This will result in mapping the literature to allow an overview and description of the methods, and provide a platform for future studies.

Alongside studies on awareness, the review will include studies using alternative terminology of “anosognosia”, “insight”, “denial”, "unawareness", and “metacognition” in PwD, as these terms are also used in the literature, as previously discussed, along with studies that measure the discrepancy between self- and informant ratings of relevant issues, or discrepancy between self-rating and objective performance, where this has been interpreted as a measure of awareness. We will include studies addressing any object of awareness, e.g. awareness of memory or of activities of daily living and other objects, as well as studies that do not specify a particular object.

### Search strategy

A search of relevant, peer-reviewed, published literature will be made from the following electronic bibliographic databases: PubMed, Embase, PsycInfo, Cumulative Index to Nursing and Allied Health Literature (CINAHL) complete, Web of Science Core collection, Cochrane Library. The following search terms were developed for use from earlier pilot searches with identification of keywords from relevant articles, and in consultation with experienced researchers and librarians: (dement* OR Alzheimer* OR “Pick’s disease”) AND (aware* OR unaware* OR anosognosia OR insight OR denial OR metacognit* OR discrepanc*), with unlimited date range and no language restriction (see attached search strategy Additional file 1). We included “Pick’s disease” as an additional term (whilst excluding Niemann Pick), to find older studies of frontotemporal dementia that may have used this diagnostic label, as pilot searches showed that it was not otherwise detected using the search terms dement* and Alzheimer*. Duplicate records will be removed. Titles/abstracts will be screened for removal of irrelevant articles. The remaining full texts will be examined according to pre-arranged eligibility criteria to produce a list of the final articles to include in the review. Title and abstract screening will be performed independently by two researchers, and at full text screening, a second researcher will screen a 10% sample of the articles for selection. Any differences will be discussed and, if required, resolved by consultation with a third senior researcher. To prevent conflicts of interest, review team members who have authored reports of studies being considered for inclusion at any stage of the selection process will not be involved in decisions about the inclusion of those studies.

### Study selection

#### Population

We will include studies measuring awareness (or equivalent term) in people with a clinical diagnosis of dementia, of any type or severity of dementia. Studies will be included from community, outpatient, in-patient and residential settings, with no limitations of age, gender or ethnicity. Studies reporting on mixed populations will be included if at least 50% of the study population has the relevant clinical diagnosis of dementia and if the data for the dementia subgroup is separately identifiable.

#### Concepts

Studies that measure awareness in PwD using standardized tools or new methods devised for that study will be included. We will include studies measuring awareness as a global construct, or of specific objects, e.g. awareness of memory, functional ability, socio-emotional functioning [22] or if defined at different levels, e.g. sensory perception, performance monitoring/on-line awareness, evaluative judgement and metacognitive awareness [18].

#### Context

Study selection will focus on articles published in English, where full text is available. Quantitative, mixed method, qualitative studies and case studies will be included. Multiple reports that use data from the same study will be identified and collated, to avoid duplication of findings.

#### Exclusion criteria

- Mild cognitive impairment or non-dementia diagnosis
- Information regarding dementia participants is not separately identifiable
- Did not measure awareness construct in people with dementia
- Full text not readily available or not in English
- Review/editorials/letters to editor with no original findings
- Non-peer reviewed material
- Conference abstracts
- Errata/correction of no significance to required data

Exclusion decisions and characteristics of excluded studies will be recorded. The search findings will be summarised using a PRISMA flow diagram [39].

### Data extraction and mapping results

Standard information, agreed in team discussions after earlier pilots of data retrieval, will be recorded for each included study and charted according to key characteristics. A database will be used to record findings and may be refined during the review process.

### Collating, summarising and reporting results

Overall results will be tabulated according to the method used and population studied, including diagnosis and severity of dementia, setting, type of study and definitions used and object of awareness studied. This will show the breadth of methods used, leading to a basic numerical summary. In keeping with published scoping review guidance [36–38], we will not formally assess quality of included studies. Themes will be explored, with a focus on describing the characteristics and utility of the methods, and identifying standardized tools, in keeping with the research objectives. This will be an iterative process, leading to a narrative report of the findings. Consultation with experts in the field will be considered at this stage, to add validity to the findings and help inform future research.

### Dissemination and ethics

The completed review will be written up and submitted for journal publication. In addition, preliminary findings will be presented as a conference poster. Ethical approval is not required for this study.

## Discussion

This scoping review will provide an overview of the methods used to measure awareness in people with dementia, allowing comparison of the methods and recognition of gaps in research. It will also identify validated measures, to allow further appraisal of the properties of these measures or components, in consideration of potential clinical use. Acknowledging gaps in research, and strengths and limitations of methods employed, will help direct further research in this area.

## Additional file

Additional file 1: Search strategy used for Embase and PsycInfo. (PDF 85 kb)

## Data Availability

Data sharing is not applicable to this article as no datasets will be generated or analysed during the current study. Search strategy for Embase and PsycInfo available in Additional file 1.

## Abbreviations

CINAHL: Cumulative Index to Nursing and Allied Health Literature
PRISMA: Preferred Reporting Items for Systematic Reviews and Meta-Analyses
PwD: People with dementia

## References

[1] World Health Organization. International statistical classification of diseases and related health problems (11th Revision). Retrieved from https://icd.who.int/browse11/l-m/en. 2018. Accessed 28 Mar 2019.

[2] American Psychiatric Association. Diagnostic and Statistical Manual of Mental Disorders. 5th ed. Washington, DC; 2013.

[3] Mograbi DC, Ferri CP, Sosa AL, Stewart R, Laks J, Brown R, Morris RG (2012). Unawareness of memory impairment in dementia: a population-based study. Int Psychogeriatr.

[4] Starkstein SE (2014). Anosognosia in Alzheimer’s disease: diagnosis, frequency, mechanism and clinical correlates. Cortex.

[5] Parrao T, Brockman S, Bucks RS, Bruce DG, Davis WA, Hatch KK, Leavy TL, Axten CA, Starkstein SE (2017). The Structured Interview for Insight and Judgment in Dementia: Development and validation of a new instrument to assess awareness in patients with dementia. Alzheimers Dement (Amst).

[6] Marková IS, Clare L, Whitaker CJ, Roth I, Nelis SM, Martyr A, Roberts JL, Woods RT, Morris R (2014). Phenomena of awareness in dementia: heterogeneity and its implications. Conscious Cogn.

[7] Martyr A, Clare L (2018). Awareness of functional ability in people with early-stage dementia. Int J Geriatr Psychiatry.

[8] Clare L, Whitaker CJ, Nelis SM, Martyr A, Markova IS, Roth I, Woods RT, Morris RG (2011). Multidimensional assessment of awareness in early-stage dementia: a cluster analytic approach. Dement Geriatr Cogn Disord.

[9] DeBettignies BH, Mahurin RK, Pirozzolo FJ (1990). Insight for impairment in independent living skills in Alzheimer’s disease and multi-infarct dementia. J Clin Exp Neuropsychol.

[10] Nelis SM, Clare L, Martyr A, Marková IS, Roth I, Woods RT, Whitaker CJ, Morris RG (2011). Awareness of social and emotional functioning in people with early-stage dementia and implications for carers. Aging Ment Health.

[11] Martyr A, Nelis SM, Quinn C, Wu Y-T, Lamont RA, Henderson C, Clarke R, Hindle JV, Thom JM, Jones IR (2018). Living well with dementia: a systematic review and correlational meta-analysis of factors associated with quality of life, well-being and life satisfaction in people with dementia. Psychol Med.

[12] Clare L, Wilson BA, Carter G, Roth I, Hodges JR (2004). Awareness in early-stage Alzheimer’s disease: relationship to outcome of cognitive rehabilitation. J Clin Exp Neuropsychol.

[13] Babinski J (1914). Contribution a l'etude des troubles mentaux dans l'hemiplegie organique cerebrale (anosognosie). Contribution to the study of the mental disorders in hemiplegia of organic cerebral origin (anosognosia). Rev Neurol.

[14] Starkstein SE, Jorge R, Mizrahi R, Robinson RG (2006). A diagnostic formulation for anosognosia in Alzheimer's disease. J Neurol Neurosurg Psychiatry.

[15] Markova IS, Berrios GE (2006). Approaches to the assessment of awareness: conceptual issues. Neuropsychol Rehabil.

[16] Weinstein EA, Friedland RP, Wagner EE (1994). Denial/unawareness of impairment and symbolic behavior in Alzheimer’s disease. Neuropsychiatry Neuropsychol Behav Neurol.

[17] Sunderaraman P, Cosentino S (2017). Integrating the constructs of anosognosia and metacognition: a review of recent findings in dementia. Curr Neurol Neurosci Rep.

[18] Clare L, Marková IS, Roth I, Morris RG (2011). Awareness in Alzheimer’s disease and associated dementias: theoretical framework and clinical implications. Aging Ment Health.

[19] Martyr A, Clare L, Nelis SM, Roberts JL, Robinson JU, Roth I, Marková IS, Woods RT, Whitaker CJ, Morris RG (2011). Dissociation between implicit and explicit manifestations of awareness in early stage dementia: evidence from the emotional Stroop effect for dementia-related words. Int J Geriatr Psychiatry.

[20] Mograbi DC, Morris RG (2013). Implicit awareness in anosognosia: clinical observations, experimental evidence, and theoretical implications. Cogn Neurosci.

[21] Clare L (2010). Awareness in people with severe dementia: review and integration. Aging Ment Health.

[22] Marková IS, Clare L, Wang M, Romero B, Kenny G (2005). Awareness in dementia: conceptual issues. Aging Ment Health.

[23] Turro-Garriga O, Garre-Olmo J, Lopez-Pousa S, Vilalta-Franch J, Rene-Ramirez R, Conde-Sala JL (2014). Abridged scale for the screening anosognosia in patients with dementia. J Geriatr Psychiatry Neurol.

[24] Clare L, Whitaker R, Quinn C, Jelley H, Hoare Z, Woods B, Downs M, Wilson B (2012). AwareCare: development and validation of an observational measure of awareness in people with severe dementia. Neuropsychol Rehabil.

[25] Reed BR, Jagust WJ, Coulter L (1993). Anosognosia in Alzheimer’s disease: relationships to depression, cognitive function, and cerebral perfusion. J Clin Exp Neuropsychol.

[26] Smith CA, Henderson VW, McCleary CA, Murdock GA, Buckwalter JG (2000). Anosognosia and Alzheimer’s Disease: The role of depressive symptoms in mediating impaired insight. J Clin Exp Neuropsychol.

[27] Snow AL, Norris MP, Doody R, Molinari VA, Orengo CA, Kunik ME (2004). Dementia Deficits Scale. Rating self-awareness of deficits. Alzheimer Dis Assoc Disord.

[28] Migliorelli R, Tesón A, Sabe L, Petracca G, Petracchi M, Leiguarda R, Starkstein SE (1995). Anosognosia in Alzheimer’s disease: a study of associated factors. J Neuropsychiatry Clin Neurosci.

[29] Clare L, Marková IS, Verhey F, Kenny G (2005). Awareness in dementia: a review of assessment methods and measures. Aging Ment Health.

[30] Lacerda IB, Sousa MFB, Santos RL, Nogueira MML, Dourado MCN (2016). Concepts and objects of awareness in Alzheimer’s disease: an updated systematic review. J Bras Psiquiatr.

[31] Brandt M, de Carvalho RLS, Belfort T, Dourado MCN (2018). Metamemory monitoring in Alzheimer’s disease A systematic review. Dement Neuropsychol.

[32] Wilson RS, Sytsma J, Barnes LL, Boyle PA (2016). Anosognosia in dementia. Curr Neurol Neurosci Rep.

[33] Moher D, Shamseer L, Clarke M, Ghersi D, Liberati A, Petticrew M, Shekelle P, Stewart LA, Group P-P (2015). Preferred reporting items for systematic review and meta-analysis protocols (PRISMA-P) 2015 statement. Syst Rev.

[34] Moher D, Stewart L, Shekelle P (2016). Implementing PRISMA-P: recommendations for prospective authors. Syst Rev.

[35] Tricco AC, Lillie E, Zarin W, O'Brien KK, Colquhoun H, Levac D, Moher D, Peters MDJ, Horsley T, Weeks L (2018). PRISMA Extension for Scoping Reviews (PRISMA-ScR): Checklist and explanation. Ann Intern Med.

[36] Arksey H, O'Malley L (2005). Scoping studies: towards a methodological framework. Int J Soc Res Methodol.

[37] Levac D, Colquhoun H, O'Brien KK (2010). Scoping studies: advancing the methodology. Implement Sci.

[38] Joanna Briggs Institute. The Joanna Briggs Institute Reviewers’ manual 2015: methodology for JBI scoping reviews. Adelaide: The Joanna Briggs Institute; 2015.

[39] Moher D, Liberati A, Tetzlaff J, Altman DG, Group P (2009). Preferred reporting items for systematic reviews and meta-analyses: the PRISMA statement. PLoS Med.

