# Supplementary material for: Measuring awareness in people with dementia: protocol for a scoping review
Source: Syst Rev. 2019 Jul 4;8:160. doi: 10.1186/s13643-019-1078-5 (PMC6610918; doi:10.1186/s13643-019-1078-5)
Supplement: Supplementary file 1 — Search strategy used for Embase and PsycInfo. (PDF 85 kb) [file 13643_2019_1078_MOESM1_ESM.pdf]

Search strategy for Embase and PsycInfo searches; scoping review

1. dement*.ti. or dement*.ab.

2. alzheimer*.ti. or alzheimer*.ab.

3. 1 or 2

4. (("pick's disease" or "picks disease" or "pick disease") not ("niemann-pick" or "niemann pick")).ti. or (("pick's disease" or "picks disease" or "pick disease") not ("niemann-pick" or "niemann pick")).ab.

5. 3 or 4

6. aware*.ti. or aware*.ab.

7. 5 and 6

8. unaware*.ti. or unaware*.ab.

9. 5 and 8

10. anosognosia.ti. or anosognosia.ab.

11. 5 and 10

12. insight.ti. or insight.ab.

13. 5 and 12

14. denial.ti. or denial.ab.

15. 5 and 14

16. metacognit*.ti. or metacognit*.ab.

17. 5 and 16

18. discrepanc*.ti. or discrepanc*.ab.

19. 18 and 5

20. 7 or 9 or 11 or 13 or 15 or 17 or 19
